# Laparoscopic Nephrectomy with Adrenalectomy for Synchronous Adrenal Myelolipoma and Renal Cell Carcinoma

**DOI:** 10.1155/2015/635072

**Published:** 2015-04-29

**Authors:** Kallappan Senthil, Manickam Ramalingam, Karpagam Janardhan, Anandan Murugesan, Mizar Ganapathy Pai

**Affiliations:** ^1^Urology Clinic, Coimbatore 641018, India; ^2^Department of Urology, PSG Institute of Medical Sciences and Research, Peelamedu, Coimbatore 641025, India; ^3^GKNM Hospitals, Coimbatore 641018, India

## Abstract

*Introduction*. Adrenal myelolipomas are uncommon nonfunctioning tumors of the adrenal. Synchronous renal cell carcinomas with adrenal myelolipomas are very rare. We present the case report of adrenal myelolipoma with synchronous RCC managed laparoscopically. *Case Report*. A 60-year-old old gentleman presented with incidental right upper polar mass with right adrenal mass. Metastatic work-up was negative. Laparoscopic radical nephrectomy with adrenalectomy was done under general anesthesia. The biopsy report was right kidney clear cell adenocarcinoma (T1b) with right adrenal myelolipoma. *Conclusion*. This is the first case report of laparoscopic adrenalectomy with nephrectomy for ipsilateral synchronous renal cell carcinoma with adrenal myelolipoma.

## 1. Introduction

Adrenal myelolipomas are uncommon nonfunctioning tumours of the adrenal gland. They are usually found incidentally [1]. Myelolipoma was first described by Gierke in 1905 [2]. Adrenal adenomas with synchronous renal cell carcinomas are not uncommon [3].

However, synchronous adrenal myelolipoma associated with ipsilateral RCC is extremely rare with very few cases reported [4–6]. These were managed by open approach. We present the case report of adrenal myelolipoma with synchronous RCC managed laparoscopically.

## 2. Case Report

**Figure 1:**
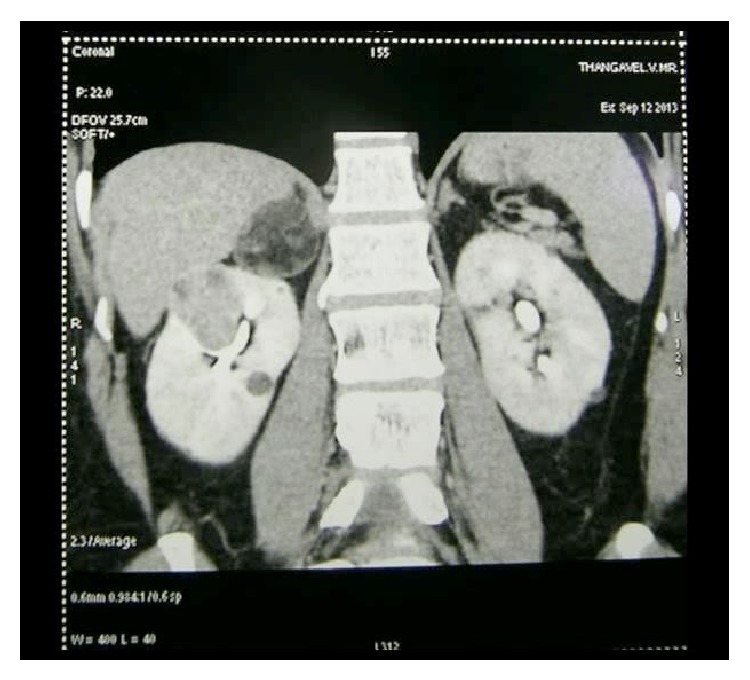
CECT scan showing right renal and adrenal tumors.

A 60-year-old gentleman presented with incidental right adrenal and renal tumor on ultrasound evaluation. General and systemic examinations were within normal limits. He was a hypertensive on treatment for 10 years. He had no other comorbidities. Blood and urine evaluations were normal. Contrast CT showed a 4 cm upper polar enhancing mass in the right kidney and a well-circumscribed 4 cm adrenal mass with fat components predominating (Figure 1).

Metastatic work-up was negative. Though adrenal tumors associated with RCCs are more commonly metastasis, the fat element in the adrenal mass suggested otherwise. He was planned for right nephrectomy with adrenalectomy. Under GA, with the patient in the right lateral position, laparoscopy was done transperitoneally.

**Figure 2:**
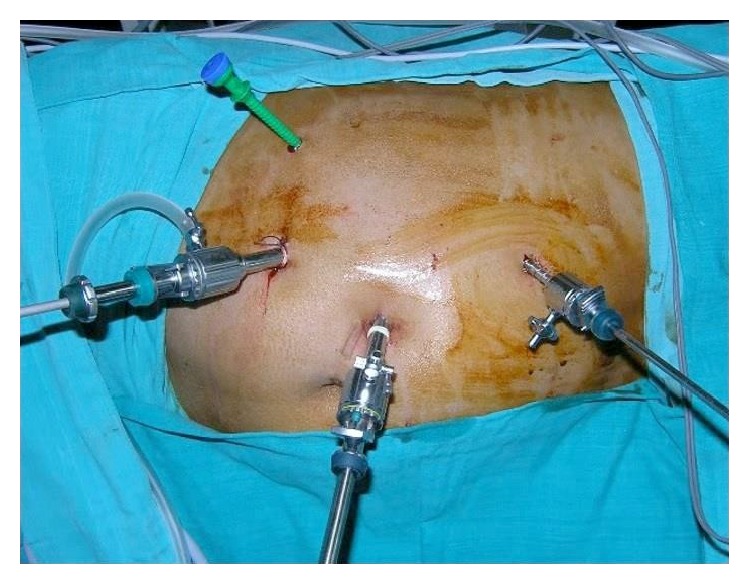
Ports position.

**Figure 3:**
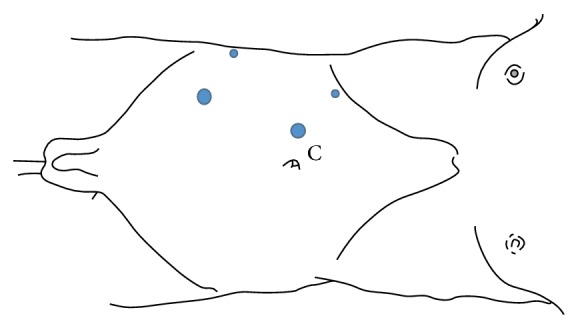
Line diagram of ports position.

Using 4 ports (Figures 2 and 3), the hepatic flexure of the colon was mobilized and the liver was retracted superiorly to visualize the mass.

The kidney was mobilized all around along with the adrenal mass. Renal artery and vein were identified, clipped, and divided. The ureter was clipped and divided. Right adrenal vein was clipped and divided. Adrenal gland was dissected superiorly and right kidney and adrenal were removed. Port sites were closed and a drain was placed. The patient had uneventful postoperative recovery.

**Figure 4:**
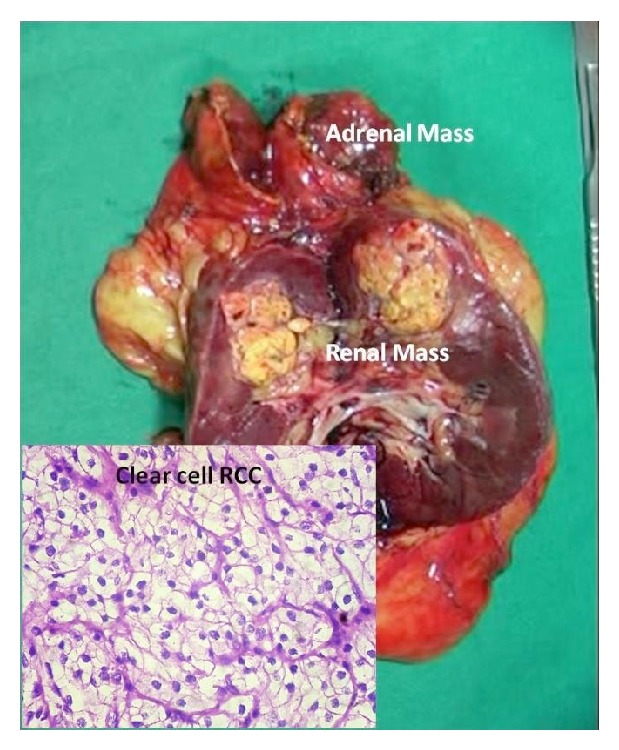
Resected specimen of the right kidney and adrenal with renal tumor microscopy.

The histopathology was right conventional clear cell renal cell carcinoma (Figure 4) confined to Gerota's fascia with no vascular invasion or lymphatic metastasis of Fuhrman grade 2 (T1b).

**Figure 5:**
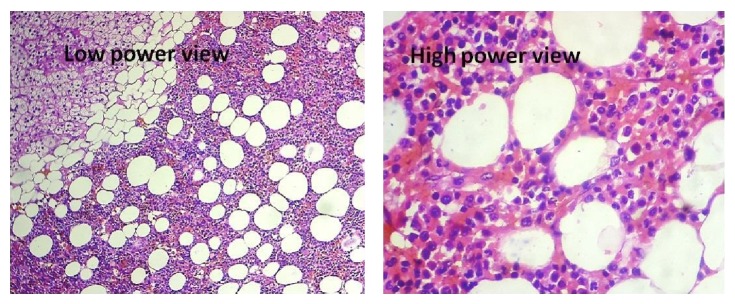
Histopathology of the adrenal myelolipoma—low and high power views.

The adrenal tumor shows a neoplasm with proliferation of lymphocytes with round to polyhedral cells with clear cytoplasm and eccentric nuclei along with marrow elements composed of myeloid precursors with few norm oblasts and megakaryocytes. These findings were consistent with myelolipoma (Figure 5).

## 3. Discussion

Adrenal myelolipomas are rare tumours arising from the adrenal. They form 2-3% of all adrenal tumours [7]. Myelolipomas contain adipose tissue with hematopoietic elements. These hematopoietic elements are formed from reticuloendothelial stem cell rests in the adrenal. The most widely accepted etiologic factor is adrenocortical cell metaplasia in response to stimuli, such as necrosis, inflammation, infection, or stress [8]. They may be associated with other adrenal benign or malignant tumours [7]. Renal tumours are associated with adrenal tumors in many instances. In a study done by Bahrami et al. among 550 cases of radical nephrectomy with ipsilateral adrenalectomy, 80 cases of coexisting renal and adrenal masses were identified [3]. Most of them were metastatic tumors or nonfunctioning adenomas. Only few case reports of renal cell carcinoma associated with ipsilateral myelolipoma are published [4–6]. Though adrenal myelolipomas are managed laparoscopically [9], this is the first case report of laparoscopic management of synchronous ipsilateral adrenal myelolipoma with renal cell carcinoma. Myelolipomas can be observed if they are asymptomatic [7]. Sometimes myelolipomas can produce retroperitoneal hemorrhage necessitating emergency intervention. Larger myelolipomas more than 4–6 cm need excision as they are more prone to such complications [10]. Bilateral myelolipomas have also been reported [11]. With the wide spread use of laparoscopy, laparoscopic adrenalectomy is preferred for adrenal tumours, with the benefits of minimally invasive approach [12, 13].

## 4. Conclusion

This case report illustrates and discusses the first case of laparoscopic adrenalectomy with nephrectomy for ipsilateral synchronous renal cell carcinoma with adrenal myelolipoma in literature. Laparoscopic nephrectomy with adrenalectomy for ipsilateral synchronous adrenal myelolipoma and renal cell carcinoma is a safe and feasible procedure.
